# Oral L-citrulline supplementation enhances cycling time trial performance in healthy trained men: Double-blind randomized placebo-controlled 2-way crossover study

**DOI:** 10.1186/s12970-016-0117-z

**Published:** 2016-02-19

**Authors:** Takashi Suzuki, Masahiko Morita, Yoshinori Kobayashi, Ayako Kamimura

**Affiliations:** Healthcare Products Development Center, KYOWA HAKKO BIO CO., LTD., 2, Miyukigaoka, Tsukuba, Ibaraki 305-0841 Japan; Laboratory of Pharmacognosy, School of Pharmaceutical Sciences, Kitasato University, 5-9-1, Shirokane, Minato-ku, Tokyo, 108-0861 Japan

**Keywords:** Ergogenic, Human, L-Citrulline, Nitric oxide (NO), Sport performance

## Abstract

**Background:**

Many human studies report that nitric oxide (NO) improves sport performance. This is because NO is a potential modulator of blood flow, muscle energy metabolism, and mitochondrial respiration during exercise. L-Citrulline is an amino acid present in the body and is a potent endogenous precursor of L-arginine, which is a substrate for NO synthase. Here, we investigated the effect of oral L-citrulline supplementation on cycling time trial performance in humans.

**Methods:**

A double-blind randomized placebo-controlled 2-way crossover study was employed. Twenty-two trained males consumed 2.4 g/day of L-citrulline or placebo orally for 7 days. On Day 8 they took 2.4 g of L-citrulline or placebo 1 h before a 4-km cycling time trial. Time taken to complete the 4 km cycle, along with power output/VO_2_ ratio (PO/VO_2_), plasma nitrite and nitrate (NOx) and amino acid levels, and visual analog scale (VAS) scores, was evaluated.

**Results:**

L-Citrulline supplementation significantly increased plasma L-arginine levels and reduced completion time by 1.5 % (*p* < 0.05) compared with placebo. Moreover, L-citrulline significantly improved subjective feelings of muscle fatigue and concentration immediately after exercise.

**Conclusions:**

Oral L-citrulline supplementation reduced the time take to complete a cycle ergometer exercise trial.

**Trial registration:**

Current Controlled Trials UMIN000014278.

## Background

NO plays key roles such as maintaining the function and integrity of the endothelium, including vascular tone and structure [1]. In sports physiology, nitrate supplementation is thought to be an ergogenic aid [2–4]. This view is based on evidence that NO is an important modulator of blood flow and mitochondrial respiration under physiological conditions [5]. Some studies have shown that dietary NO related supplements, such as nitrate-rich beetroot juice, enhance human sport performance [6–9]. Dietary supplementation with nitrate thus appears to be beneficial for exercise.

There is growing interest in the use of L-citrulline as an NO-related dietary ingredient. L-Citrulline is present in the body and is a potent endogenous precursor of L-arginine [10], which is a substrate for NO synthase (NOS). NOS catalyzes a complex enzymatic reaction that leads to NO formation from L-arginine and oxygen and generates L-citrulline as a byproduct [11]. L-Citrulline is effectively recycled via the L-citrulline NO cycle to L-arginine and plays an important role in the metabolism and regulation of NO [12]. L-Citrulline supplementation has various beneficial effects, such as ameliorating arterial stiffness [13] and improving erectile function [14], memory [15], O_2_ uptake kinetics, and high-intensity exercise performance [16] through upregulation of NO synthesis. We and others have demonstrated in animal models that oral supplementation with L-citrulline upregulates endothelial NO synthase (eNOS) expression, improves endothelial function, and plays an atheroprotective role [17–19]. Interestingly, a clinical trial has shown that oral intake of L-citrulline dose-dependently and more effectively increases plasma L-arginine levels than does L-arginine supplementation in healthy human volunteers [20]. Therefore, L-citrulline may be considered an effective L-arginine and NO supplies which might be expected to have potential for enhancing sport performance. Some studies have found that acute L-citrulline supplementation has no effect on exercise [21, 22]. On the other hand, Bailey et al. [16] showed that 6 days of L-citrulline supplementation improved exercise tolerance. This suggests that chronic L-citrulline supplementation (for about 1 week) is needed to enhance exercise tolerance. However, it is not presently known whether chronic small doses of L-citrulline enhance sport performance. Moreover, to our knowledge, no study has comprehensively evaluated the effects of L-citrulline on endurance exercise performance during simulated competition or on subjective feelings of discomfort associated with exercise in humans. We hypothesized that chronic L-citrulline administration would enhance performance during simulated competition.

The aim of this study was to investigate the effect of oral supplementation of L-citrulline on cycling time-trial performance in healthy trained men.

## Methods

### Subjects

Twenty-five trained healthy Japanese males volunteered to take part in this double-blind, randomized, placebo-controlled, two-way crossover trial. Because of the crossover design, half of the subjects participated under one condition and half under the other at the same time, with a washout period of 3 weeks. Randomization was conducted by using SAS 9.3 (SAS Institute Inc.). The subjects recruited were aged 20 to 39 years and participated in sport twice a week or more. The sports included athletics (long distance running), baseball, cycling, soccer, triathlon, and skiing. Current smokers, subjects taking medication or dietary supplements for chronic conditions, and subjects with injuries that could interfere with their performance were excluded. The participants’ health status was assessed by both physical and laboratory examinations, including an electrocardiogram and blood chemistry panel. Three males were excluded from the analysis because they had colds on the test day. We therefore analyzed a final total of 22 males (mean ± SD age, 29 ± 8.4 years; body mass, 74 ± 9.4 kg; height, 175 ± 4.1 cm; body mass index, 24 ± 3.3 kg/m^2^). The subjects were instructed not to change their usual training volume or diet during the 7 days of the study. The protocol was conducted according to the Declaration of Helsinki and was approved by the Ethics Committee of Fukuda Clinic (Osaka, Japan). All subjects gave their written informed consent.

### Study design

**Fig. 1:**
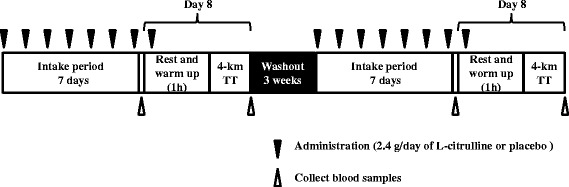
Study design

The study was conducted at Fukuda Clinic (Osaka, Japan). After enrollment, the subjects were randomized into two groups to receive the following treatments once a day for 1 week before the experimental day in a double-blind fashion: 9 capsules consisting of 2.4 g of L-citrulline (KYOWA HAKKO BIO CO., Ltd., Tokyo, Japan) or 9 placebo capsules consisting of 2.4 g of cornstarch (Nippon NSC Co., Ltd., Tokyo, Japan) before bedtime. The indistinguishability of the capsules was confirmed by the Ethics Committee of Fukuda Clinic (Osaka, Japan). The purity of L-citrulline was analyzed by using an amino acid analyzer (L-8900 Hitachi High-Technologies Corporation, Tokyo, Japan) [23, 24]. There is no recommended dose for L-citrulline intake to enhance sport performance, but a dose of 6 or 8 g of L-citrulline malate has been used in other studies [25, 26]. On the day before each test day, subjects were all given the same meals, which they were required to finish by 9:00 PM; they then fasted overnight. The following morning, blood pressure and heart rate were measured and blood samples were collected. Thereafter, the subjects had breakfast (a rice ball, about 180 kcal) to provide energy 1 h before intake of the 9 capsules of placebo or L-citrulline. After the intake of placebo or L-citrulline, the subjects rested quietly before takingpart in a 4-km cycling time trial (TT). Before the TT, each subject completed a warm-up. The TT was performed on a cycle ergometer (Aerobike 75XL2; Konami Sports & Life Co., Ltd., Tokyo, Japan) [27] 1 h after of the intake of placebo or L-citrulline. After the TT, blood samples were collected from the brachial vein. The study design is summarized in Fig. 1.

### Physical working capacity test

Subjects completed a physical working capacity test to determine work rate during the TT. Physical working capacity is an index employed in performance diagnostics to appraise the tested person’s aerobic performance capacity. The protocol began with 3 min of 25-watt (W) cycling, after which 3 min each of 75-W and 125-W cycling was imposed. Physical working capacity at 75 % of the predicted maximum heart rate (PWC_75%HRmax_) was determined from the relationship between HR in the final 30 s and exercise work load in the above-mentioned incremental exercise test. PWC_75%HRmax_ was evaluated as work load at 75 % of HR_max_ (=220 – age) [28]. The subjects were then familiarized with the cycle ergometer.

#### Time trial test

On Day 8, one hour after intake of 2.4 g of placebo or L-citrulline, each subject performed the TT. The work rate of each subject was set at 60 rpm and PWC_75%HRmax_. Time to complete 4 km of cycling, power output (PO), VO_2_, plasma nitrite and nitrate (NOx) levels, plasma amino acid concentrations, and visual analog scale (VAS) scores were evaluated. The computrainer ergometry system re**c**orded PO every 10 s, and these values were averaged for every 0.5 km completed in the TT to create a PO profile. During the TT, breath-by-breath pulmonary gas exchange and ventilation were measured continuously (Aero Monitor AE-300S, Minato Medical Science Co., Ltd., Osaka, Japan) [29–31]. These data were also used to produce a PO/VO_2_ ratio, namely the PO produced in watts per liter of O_2_ consumed per min (W/L/min).

#### Visual analog scale

Subjects were asked to subjectively rate their degree of discomfort on a VAS from 0 mm (excellent) to 100 mm (poor) after the TT. The VAS was originally developed for measuring pain level [32] and has also been used to assess fatigue level [33].

#### Blood sample analyses

Blood samples were collected from the brachial vein; 5 mL was collected each time. Plasma samples were prepared by collecting blood in an EDTA-2Na-containing tube and kept on ice until centrifugation at 1700 *g* for 10 min at 4 °C. Plasma NOx was assayed via the Griess reaction by using a colorimetric assay kit (Nitrate/Nitrite Colorimetric Assay Kit, Cayman, Ann Arbor, MI, USA) [34, 35]. To assess amino acids, the plasma sample was deproteinized with 4 % sulfosalicylic acid (plasma to 20 % sulfosalicylic acid ratio = 0.3: 0.075 mL) for 30 min on ice and then centrifuged at 1700 *g* for 10 min at 4 °C. The supernatant was stored at −80 °C until analysis. The concentrations of amino acids (L-valine, L-isoleucine, L-leucine, L-arginine, and L-citrulline) in the plasma were measured with an amino acid analyzer (L-8900 Hitachi High-Technologies Corporation, Tokyo, Japan) [23, 24].

#### Statistical analyses

Values are shown as means ± S.E.M. Paired *t*-tests were used to evaluate the significance of any differences between the placebo and l-citrulline groups. Analyses were performed with SPSS Statistics 22 (IBM Japan, Ltd., Tokyo, Japan). *P* values of below 0.05 were regarded as statistically significant.

## Results

### Blood chemistry

**Fig. 2:**
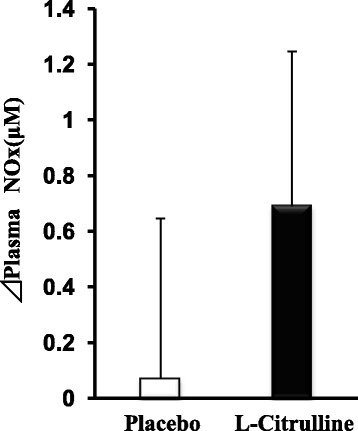
Changes in plasma NOx before and after a 4 km TT. Values are means ± S.E.M. *n* = 22

Plasma amino acid concentrations are summarized in Table 1. Seven days’ intake of L-citrulline significantly increased the plasma L-arginine level. On Day 8, plasma L-citrulline and L-arginine levels after TT were significantly higher in the l-citrulline group than in the placebo group. Levels of plasma branched chain amino acids (BCAAs: L-valine, L-isoleucine, L-leucine) were significantly lower at pre-load and post-load in the l-citrulline group than in the placebo group. There was no significant difference in the level of plasma NOx between the placebo and L-citrulline groups (Fig. 2).

**Table 1:** Plasma amino acid concentration on Day 8

	before TT and before intake		after TT and after intake	
	Placebo	L-Citrulline	Placebo	L-Citrulline
L-Citrulline (nmol/ml)	39.3 ± 1.4	54.3 ± 11.0	40.0 ± 1.4	475 ± 37^**^
L-Arginine (nmol/ml)	110 ± 4	139 ± 7^**^	110 ± 4	192 ± 9^**^
BCAA (nmol/ml) (valine, isoleucine, leucine)	565 ± 15	553 ± 21^*^	518 ± 20	501 ± 15^*^

Values are means ± S.E.M. *n* = 22, ^*^ *p* < 0.05, ^**^ *p* < 0.001, indicating a significant difference from placebo

### Physical performance test

**Fig. 3:**
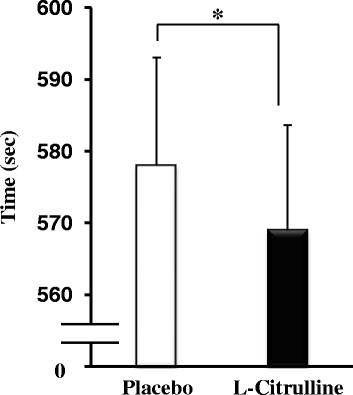
Completion times of the 4 km TT after placebo or L-citrulline supplementation. Values are means ± S.E.M. *n* = 22, * *p* < 0.05, indicating a significant difference from placebo

**Fig. 4:**
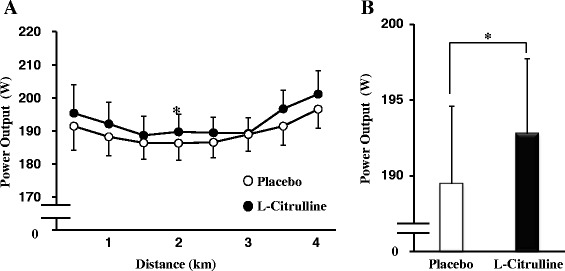
PO profile during the 4 km TT (**a**) and mean PO (**b**) of the 4 km TT after placebo or L-citrulline supplementation. Values are means ± S.E.M. *n* = 22. * *p* < 0.05, indicating a significant difference from placebo

**Fig. 5:**
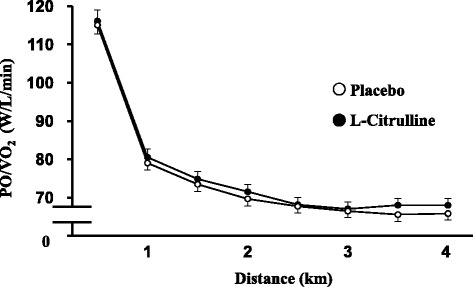
PO/VO_2_ during the 4 km TT after placebo or L-citrulline supplementation. Values are means ± S.E.M. *n* = 22

Mean TT completion times are displayed in Fig. 3, and the accompanying PO profiles are shown in Fig. 4a. L-Citrulline supplementation significantly reduced completion time compared with placebo, with a group mean reduction of 1.5 % (placebo: 578 ± 15 s, L-citrulline: 569 ± 14 s, *p* < 0.05, Fig. 3). Ingestion of L-citrulline increased mean PO by 2 % (placebo = 189 ± 5 W vs. L-citrulline = 193 ± 5 W, *p* < 0.05, Fig. 4b). There was no significant difference in VO_2_ response between placebo and L-citrulline (Table 2). PO/VO_2_ tended to be higher in the L-citrulline-supplemented group in three of six elapsed distances (*p* < 0.1, Fig. 5).

**Table 2:** VO_2_ profile during the 4 km TT

Distance (km)	Placebo	L-Citrulline
	VO_2_ (mL/min)	VO_2_ (mL/min)
0.0 ~ 0.5	1662 ± 236	1682 ± 255
0.5 ~ 1.0	2396 ± 311	2400 ± 341
1.0 ~ 1.5	2553 ± 319	2539 ± 386
1.5 ~ 2.0	2692 ± 353	2676 ± 381
2.0 ~ 2.5	2777 ± 346	2801 ± 369
2.5 ~ 3.0	2861 ± 344	2847 ± 363
3.0 ~ 3.5	2933 ± 361	2911 ± 377
3.5 ~ 4.0	3005 ± 381	2971 ± 397

Values are means ± S.E.M. *n* = 22

### Visual analog scale

**Fig. 6:**
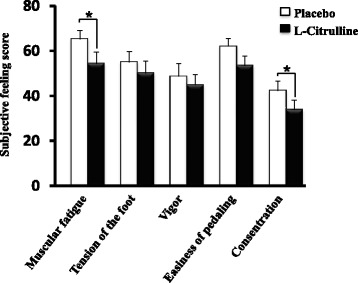
Subjective feelings immediately after exercise. Values are means ± S.E.M. *n* = 22. * *p* < 0.05, indicating a significant difference from placebo

L-Citrulline significantly improved subjective feelings of muscle fatigue, and concentration, immediately after exercise (Fig. 6). A marked but not statistically significant improvement in ease of pedaling was observed with L-citrulline supplementation (*p* < 0.1).

## Discussion

We demonstrated that oral supplementation with L-citrulline at 2.4 g/day for 7 days significantly increased plasma L-arginine levels. Moreover, intake of L-citrulline for 7 days and I h before the TT significantly increased plasma L-citrulline and L-arginine levels and enhanced cycling TT performance. In addition, subjective feelings of muscle fatigue, and concentration, right after exercise were significantly improved with L-citrulline.

In this human trial, the subjects engaged in TT cycling to allow us to evaluate their exercise performance. Competitive sports typically require athletes to complete a given distance in the shortest possible time. Time-to-exhaustion tests are primarily measures of “exercise capacity,” and because there is no competitive sports event in which competition is based on time and distance before exhaustion, tests of this type have limited physiological validity [36]. It has also been reported that there is no relationship between measured time-to-exhaustion and actual cycling performance [37]. In contrast, the TT protocol used here has a high level of physiological validity [36], provides an accurate simulation of physiological responses during competition [38], and is well correlated with actual race performance [39]. Therefore, L-citrulline intake might be expected to enhance performance in real competitive sport. We used cornstarch as the placebo and the study design was a double-blind crossover; we expected that these factors would reduce the placebo effect.

The improved TT performance after supplementation with L-citrulline was the consequence of significantly greater PO for the same VO_2_. Interestingly, there was a significant correlation between plasma NOx and PO/VO_2_ after supplementation with L-citrulline but placebo had no correlation (data not shown). This finding suggests that the effects of L-citrulline on PO/VO_2_ may have been related to improved plasma NO availability, which in turn may have enhanced sport performance. In sports nutrition, NO-related products are attracting a lot of attention for their ergogenic effects. Many applied studies in humans report that NO improves sports performance. This is because NO is a potential modulator of blood flow, muscle energy metabolism, and mitochondrial respiration during exercise [5, 6, 40, 41]. Dietary supplementation with nitrate reduces the O_2_ cost of submaximal cycling [6, 42], knee extensor exercise [43], and treadmill walking and running [44]. L-Arginine is the direct precursor of NO via NOS activity. Moreover, oral intake of L-arginine improves sports performance in healthy subjects [45–47]. However, a relatively large dose (6 to 14.2 g/day) of L-arginine would be required for beneficial effects on sports performance, because L-arginine is degraded by arginase in the small intestine and liver [48–50]. For this reason, some studies have shown no effect of L-arginine on O_2_ cost and sports performance [51, 52]. In contrast, L-citrulline is not metabolized in the intestine or liver [53]. On entering the kidneys, vascular endothelium, and other tissues, L-citrulline is readily converted to L-arginine, thus raising plasma and tissue levels of L-arginine and enhancing NO production [10]. We found here that oral intake of L-citrulline increased not only L-citrulline levels but also L-arginine levels. It has been reported that oral supplementation with L-citrulline increases plasma L-arginine levels more effectively than does L-arginine supplementation in healthy subjects [20], and increased plasma L-arginine levels before exercise enhance sport performance [16]. However, some studies have found that oral L-citrulline supplementation has no effect on exercise [21, 22]. This is likely because a single dose of L-citrulline is insufficient to enhance sport performance. Bailey et al. [16] demonstrated that 6 days of L-citrulline supplementation improved exercise tolerance. These findings suggest that L-citrulline needs to be taken continuously (for about 1 week) to enhance exercise tolerance. This is why we conducted an 8-day trial, which showed positive effects of L-citrulline. The daily dose of L-citrulline in our study was 2.4 g. This seems smaller than those used in other previous studies [16, 21, 22], but 2 to 3 g of oral L-citrulline has been reported to increase plasma L-arginine levels [20, 54]. Moinard et al. [54] showed that the C_max_ of plasma L-arginine was 146 μM when subjects consumed 2 g of L-citrulline. In the study by Bailey et al. [16], the L-citrulline group had a mean plasma L-arginine level of 135 μM and showed improved exercise performance. Therefore, we had hypothesized that 2.4 g/day of L-citrulline for 8 days might be enough to increase plasma L-arginine levels such that we would obtain ergogenic effects. In fact, Bailey et al. reported that the plasma L-arginine level in their L-citrulline group was about 2.3 times that in their placebo group [16]. On the other hand, our data demonstrated that the plasma L-arginine level in our L-citrulline group was about 1.7 times that in our placebo group. However, our absolute value of plasma L-arginine after intake of L-citrulline was higher than that in the study by Bailey et al. Therefore, 2.4 g/day of L-citrulline for 8 days is likely enough to obtain ergogenic aid.

We found here that, L-citrulline supplementation significantly increased plasma levels of plasma L-citrulline and L-arginine, which are essential for NO synthesis. In our study the plasma arginine level after supplementation of L-citrulline was as high as that in the study by Bailey et al. [16]. Our results thus suggest that L-citrulline would enhance sport performance through NO synthesis; however, we were not able to observe an increase in plasma NOx level. We measured NOx at only two time points: before and after exercise, not during exercise. These evaluation points might not have been suitable for detecting significant between-point differences in NO generation. Chemiluminescence assay is more sensitive than colorimetric assay for detecting NOx. In this study, we measured NOx by colorimetric assay, and it may not have been sensitive enough to detect changes in plasma NOx.

Sureda et al. [26] showed that oral intake of 6 g of L-citrulline malate 2 h before exercise enhances the use of BCAAs, which are metabolized in the muscles to produce energy. In our study, L-citrulline supplementation decreased plasma BCAA levels. These data indicate that L-citrulline promotes the metabolic use of these amino acids as fuel to support muscular exercise. Moreover, L-citrulline significantly improved subjective feelings of muscular fatigue. BCAA reduces muscle soreness and fatigue [55, 56]. Furthermore, L-citrulline malate reduces fatigue and post-workout muscle soreness [25]; watermelon juice, which is rich in L-citrulline, also reduces muscle soreness [57]. Our data suggest that L-citrulline has the potential to relieve muscle fatigue. Therefore, the effects of L-citrulline on BCAA utilization and muscular fatigue might also contribute to enhanced sport performance. In addition, the subjective feeling of concentration was significantly improved by oral intake of L-citrulline. Hayashi et al. have reported that L-citrulline improves blood flow [17]. The concentration-enhancing effects of L-citrulline are likely due to enhanced blood flow.

A growing number of sport supplements include L-arginine, which is claimed to enhance NO production, despite L-arginine being rapidly metabolized in the small intestine and liver when administered orally. As mentioned above, L-citrulline is a potent precursor of L-arginine, and several functional advantages of L-citrulline over L-arginine have been elucidated [17, 20]; moreover, Akashi et al. [58] have revealed that L-citrulline is an efficient hydroxyl radical scavenger. L-arginine tends to be extremely bitter and highly water absorbent, whereas L-citrulline is tasteless, odorless, and non-hygroscopic. It would thus appear that L-citrulline is superior to L-arginine in terms of ease of handling and taste as an ingredient of supplements. L-Citrulline is present in large quantities in watermelon but is not abundant in other fruits, vegetables, meat, or fish because it is a free amino acid. It is difficult to obtain L-citrulline from a conventional diet in sufficient amounts to enhance sports performance. Therefore, it may be beneficial to take a few tablets of L-citrulline before exercise as an ergogenic aid.

Our study had several limitations. We instructed the subjects not to change their training volumes and to eat their usual diets during the 7 days of the study. However, we did not make the training volumes and diets identical among the subjects, with the exception of dinner on the evening before the test day and breakfast on the test day. Some of the subjects’ performances may have been affected by intense training sessions in the 2 days before the trials. Here, we conducted a double-blind randomized placebo-controlled two-way crossover study in 22 subjects; in future, an additional, larger-scale study will be needed to verify our findings.

## Conclusions

We conclude that oral L-citrulline supplementation enhances cycling time trial performance. Moreover, L-citrulline improves subjective feelings (e.g. of muscle soreness) after performance. These data, taken together, suggest that L-citrulline is a promising amino acid for enhancing sport performance.

## Abbreviations

BCAAs: branched chain amino acids
eNOS: endothelial NO synthase
NO: nitric oxide
NOS: NO synthase
NOx: nitrite and nitrate
PO/VO_2_: power output / VO_2_ ratio
TT: time trial
VAS: Visual Analog Scale
